# Supplementary material for: Suicide prevention for international students: A single-arm mixed methods evaluation of the LivingWorks safeTALK program in Australia
Source: Glob Ment Health (Camb). 2025 Oct 24;12:e119. doi: 10.1017/gmh.2025.10082 (PMC12641313; doi:10.1017/gmh.2025.10082)
Supplement: Ng et al. supplementary material [file S2054425125100824sup001.docx]

***Introduction***

General introduction to interview: My name is [interviewer name] and I am a researcher from [university name]. For this research we are evaluating students’ experiences of the SafeTALK training for international students. During this interview, I will ask you questions about your experience. It’s important to note that there is no right or wrong answer to my questions. What will be the most helpful for me is if you give responses with as much detail as you feel comfortable sharing with me. I will guide you through the interview so that we finish it all in an hour or less and I will take handwritten notes to remind me of important ideas. Do you have any questions before we get started?

**Questions**

1. What motivated you to join the SafeTALK training?
2. Did you feel that the training program was designed with consideration for your cultural background?
3. Were the training methods used in the program consistent with your cultural learning style and preferences?
4. Did you find the training program relevant and applicable to your cultural context and experience with suicide prevention?
5. Do you feel represented by the training content?
6. What did you enjoy most about the training?
7. What did you enjoy least about the training?
8. Did you learn new things during the training?
9. Did you develop any new skills during the training?
10. Have you used knowledge or skills that you just mentioned since completing the training?
    1. How so and what was your experience of using them/why not?
11. How could the SafeTALK training be improved for international students in the future?
12. Is there anything else you think I should know before finishing up the interview?
